# A Bio‐Liposome Activating Natural Killer Cell by Illuminating Tumor Homogenization Antigen Properties

**DOI:** 10.1002/advs.202205449

**Published:** 2023-02-28

**Authors:** Xue Yang, Jiayi Bian, Zheng Wang, Mengning He, Ying Yang, Quanhao Li, Xinping Luo, Zhanwei Zhou, Jing Li, Shenghong Ju, Minjie Sun

**Affiliations:** ^1^ State Key Laboratory of Natural Medicines Department of Pharmaceutics China Pharmaceutical University Nanjing 210009 P. R. China; ^2^ Jiangsu Key Laboratory of Molecular and Functional Imaging Department of Radiology, Zhongda Hospital Medical School of Southeast University Nanjing 210009 P. R. China

**Keywords:** bio‐liposomes, cancer immunotherapy, malignant tumors, NK cells

## Abstract

Natural killer (NK) cell therapies, primarily based on chimeric antigen receptor NK cells (CAR‐NK), have been developed and applied clinically for therapeutic treatment of patients with mid‐to‐late‐stage tumors. However, NK cell therapy has limited efficacy due to insufficient antigen expression on the tumor cell surface. Here, a universal “illuminate tumor homogenization antigen properties” (ITHAP) strategy to achieve stable and controlled antigen expression on the surface of tumor cells using nanomedicine, thus significantly enhancing the immune recognizability of tumor cells, is described. The ITHAP strategy is used to generate bio‐liposomes (Pt@PL‐IgG) composed of intermingled platelet membranes and liposomes with NK‐activatable target antigen (IgG antibodies) and cisplatin pre‐drug. It is demonstrated that Pt@PL‐IgG successfully targets tumor cells using the autonomous drive of platelet membranes and achieves IgG implantation on tumor cells by utilizing membrane fusion properties. Moreover, it is shown that the Pt‐DNA complex combined with NK cell‐induced pyroptosis causes substantial interferon (IFN) secretion, thus providing a synthase‐stimulator of interferon genes (STING)‐IFN‐mediated positive immune microenvironment to further potentiate NK therapy. These results show that anchoring cancer cells with NK‐activatable target antigens is a promising translational strategy for addressing therapeutic challenges in tumor heterogeneity.

## Graphical abstract

A universal “illuminate tumor homogenization antigen properties” (ITHAP) strategy has been developed based on nanomedicines that enhance tumor immunotherapy. The prepared bio‐liposomes, called Pt@PL‐IgG, enable stable and controlled implantation of antigens on the surface of tumor cells, thereby completely increasing the immune recognizability of tumor cells. The bio‐liposomes demonstrate excellent anti‐tumor effects in various mouse models.

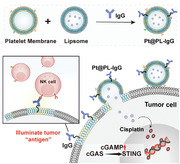

## 1 Introduction

Tumor immunotherapy is a promising therapeutic strategy for several cancers, which has recently been applied clinically.^[^ ^1^ ^]^ Natural killer (NK) cells are an important component of the body's immune system and are essential for anti‐tumor immunity,^[^ ^2^ ^]^ exerting antibody‐dependent cellular cytotoxicity (ADCC) effects to directly eliminate tumor cells.^[^ ^3^ ^]^ Moreover, recent studies have shown that NK cells induce pyroptosis in several tumors.^[^ ^4^ ^]^ Further, NK cells secrete cytokines and chemokines that recruit dendritic cells (DC) to aggregate towards solid tumors, thereby advancing the anti‐tumor immune action of CD8^+^ T cells.^[^ ^5^ ^]^ Considering these unique functional properties of NK cells, NK cell‐based anti‐cancer therapies are rapidly being developed and currently represent one of the major areas of innovation in immunotherapy, including chimeric antigen receptor NK cell (CAR‐NK) therapies and bispecific antibody therapies targeting NK cells and others.^[^ ^6^ ^]^ However, tumor heterogeneity due to variable expression of target antigens by tumor cells remains a challenge that limits the efficacy of NK therapy.^[^ ^7^ ^]^ Moreover, most tumor cells express target antigens at levels below the threshold for CAR‐NK to be effective, leading to immune escape.^[^ ^8^ ^]^ Hence, strategies to strengthen tumor homogeneity are urgently needed to develop broad‐based, stable, and controllable NK cell therapy.

Based on our in‐depth exploration of liposome and cell membrane delivery systems,^[^ ^9^ ^]^ we proposed an innovative tumor homogenization antigen properties (ITHAP) strategy to enhance the expression of tumor antigens by constructing a bio‐liposome composed of intermingled platelet membranes and liposomes, which targets specific antigens to the surface of the bio‐liposome. This strategy exploits the autonomy of platelet membranes to target tumors^[^ ^10^ ^]^ while enabling stable and controlled implantation of tumor cell surface antigens (IgG) by membrane fusion liposomes.^[^ ^11^ ^]^ Therefore, tumors with insufficient antigen expression can be precisely identified and killed using NK cells.

**Figure 1:**
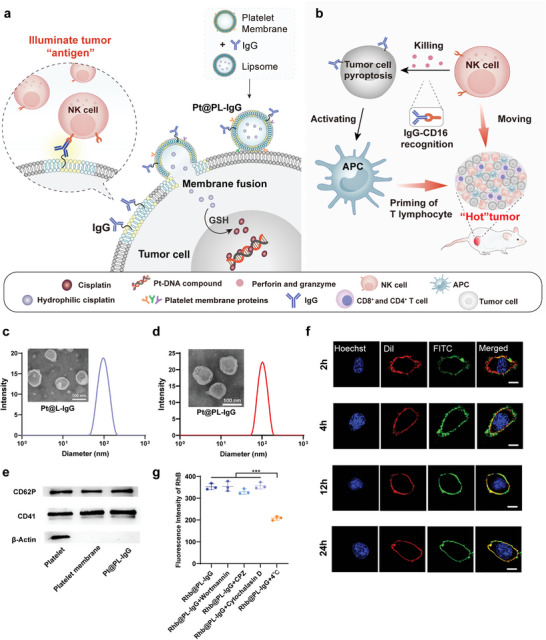
Design of the illuminate tumor homogenization antigen properties (ITHAP) strategy and characterization of Pt@PL‐IgG. a) The process of bio‐liposomes interaction with tumor cells. Following intravenous administration, Pt@PL‐IgG was recognized and adhered to the tumor cells by the platelet membranes, and fused with the tumor cells to insert NK‐activatable target antigen “IgG” onto the surface of the tumor cells. b) Following implantation of the new antigen, the tumor cells were identified by NK cells and triggered the ADCC effect. Meanwhile, the killed tumor cells activated APCs, which worked in concert with NK cells to alleviate the immunosuppressed tumor microenvironment and shape the “hot” tumor. c) Histogram of the size distribution and representative TEM images of Pt@L‐IgG. d) Histogram of the size distribution and representative TEM images of Pt@PL‐IgG. e) The expression of proteins of platelets on Pt@PL‐IgG. f) Fusogenic property of Pt@PL‐FITC evaluated using CLSM. Phospholipids were labeled with FITC (green), tumor cell membrane was labeled with DiI (red), and tumor cell nucleus was labeled with Hoechst (blue). Scale bar, 5 µm. g) Fluorescence intensity of rhodamine b (RhB) in 4T1 cells treated with PBS, wortmannin, chlorpromazine, and cytochalasin D at 37 °C and PBS at 4 °C followed by incubation with Rhb@PL‐IgG in vitro by flow cytometry, respectively (*n* = 3). Data are shown as mean ± SD; *n* represents the number of biologically independent samples. Student's *t*‐test. *P < 0.05, **P < 0.01 and ***P < 0.001 and ****P < 0.0001.

In this study, we constructed bioliposomes (Pt@PL‐IgG) carrying IgG antibodies that activate NK cells and simultaneously encapsulate cisplatin to achieve stable and controlled implantation of antigens on the surface of tumor cells to precisely initiate an immune response (**Figure** **1**a,b). Cisplatin (Pt) binds to DNA to form a complex that inhibits DNA cleavage by deoxyribonucleases^[^ ^12^ ^]^ and cooperates with NK cell‐induced pyroptosis to promote the release of Pt‐DNA complexes in tumor cells and activate the cyclic GMP‐AMP synthase‐stimulator of interferon genes (cGAS‐STING) pathway in antigen‐presenting cells (APCs), leading to substantial interferon secretion.^[^ ^13^ ^]^ Interferon reverses the immunosuppressive tumor microenvironment, further potentiating NK cell activation. Our bio‐liposomes enable in vivo self‐activation of NK cells and synergistically initiate the cGAS‐STING pathway in APCs, ameliorating poor immune efficacy. We demonstrate that this strategy has significant anti‐tumor effects in mouse models of breast cancer and disseminated non‐Hodgkin's lymphoma using bio‐liposomes, which effectively overcome tumor heterogeneity.

## 2 Results and Discussion

### 2.1 Preparation and Characterization of Bio‐Liposomes (Pt@PL‐IgG)

We developed bio‐liposomes (Pt@PL‐IgG) containing the chemotherapeutic drug cisplatin prodrug and immobilized NK activating ligands (IgG) to activate NK cells. Cisplatin was oxidized by hydrogen peroxide (H_2_O_2_) and condensed with succinic anhydride (SA) to obtain the carboxylated cisplatin prodrug (Pt(IV)‐COOH) (Figure S1 and S2, Supporting Information). Pt@PL‐IgG showed a typical liposome and vesicle structure with an appropriate particle size of 125.5 ± 3.5 nm, which is conducive for drug delivery in vivo (Figure 1c,d). Platelets contribute to malignant tumor progression by adhering to tumor cells via the integrin proteins, CD41 and CD62P,^[^ ^14^ ^]^ which are retained on Pt@PL‐IgG (Figure 1e). The particle size of the bio‐liposomes in the medium and PBS remained stable within 60 h (Figure S3, Supporting Information). Most of the platelet membrane proteins were extensively retained on Pt@PL‐IgG, as observed by SDS‐PAGE (Figure S4, Supporting Information). Moreover, bands corresponding to IgG (37 kDa) were found in Pt@PL‐IgG, indicating that IgG was successfully embedded in the bio‐liposomes membranes.

Platelets can adhere to tumor cells, which is conducive for uptake by tumor cells.^[^ ^10a^ ^]^ We used the fluorescent probe, rhodamine B (Rhb), to replace Pt in bio‐liposomes to evaluate the cellular uptake efficiency using confocal laser scanning microscopy (CLSM) and flow cytometry (Figure S5, Supporting Information). Compared to the Rhb@L‐IgG group, the Rhb@PL‐IgG group showed a significantly increased uptake efficiency, which was attributed to the introduction of the platelet membrane.

### 2.2 The Fusogenic Property of Pt@PL‐IgG

The fusogenic properties of Pt@PL‐IgG were assessed using CLSM. Briefly, phospholipids were labeled with FITC (green), the tumor cell membrane was labeled with DiI (red), and tumor cell nuclei were labeled with Hoechst (blue). We observed clear colocalization of red and green fluorescence in the cell membrane after 2 h of incubation with Pt@PL‐IgG, which lasted until 24 h (Figure 1f and Figure S6, Supporting Information), indicating that Pt@PL‐IgG was perfectly fused and stably retained on the tumor cell membrane. Subsequently, we tested the effect of different uptake inhibitors on the uptake efficiency of Pt@PL‐IgG using flow cytometry. We observed that while low temperature inhibited the cellular uptake of Pt@PL‐IgG, other uptake inhibitors, including wortmannin, chlorpromazine, and cytochalasin D, did not affect the cellular uptake efficiency, indicating that Pt@PL‐IgG did not enter cells through macropinocytosis, phagocytosis, or endocytosis (Figure 1g).^[^ ^15^ ^]^

Furthermore, we used FITC‐labeled IgG to monitor the expression of IgG on the surface of tumor cells after Pt@PL‐IgG incubation using flow cytometry and observed increased expression of IgG in tumor cells from 3.59% (PBS) to 58.7% after Pt@PL‐IgG treatment (Figure S7, Supporting Information). Moreover, Pt@PL‐IgG treatment exhibited a 2.6‐fold higher IgG expression in tumors than Pt@L‐IgG treatment, suggesting that platelet admixture is essential for a successful ITHAP strategy. Taken together, these results show that Pt@PL‐IgG enters tumor cells through membrane fusion, thereby stably expressing IgG on the tumor cell surface.

### 2.3 In Vitro Evaluation of ITHAP Strategy

**Figure 2:**
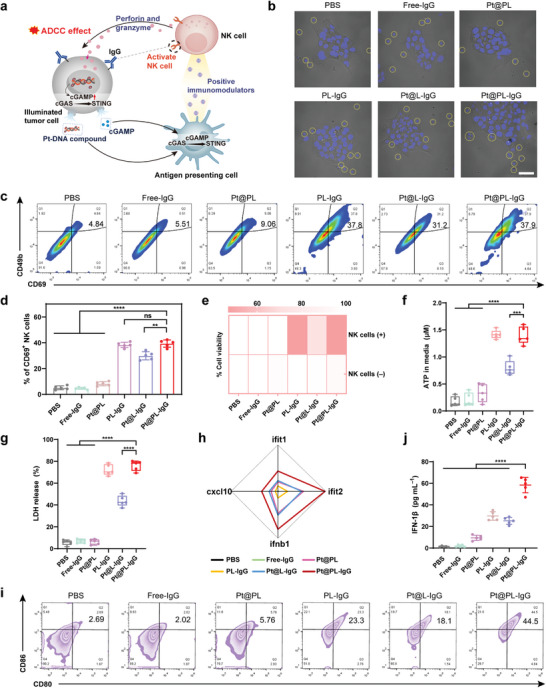
In vitro evaluation of ITHAP strategy. a) The mechanism of action of tumor cells following treatment with bio‐liposomes. NK cells recognized tumor cells after being implanted with new antigens, released perforin and granzyme to kill tumor cells, and secreted cytokines and chemokines. Simultaneously, Pt‐DNA and cGAMP were released from the tumor in concert with cisplatin and pyroptosis, activating the cGAS‐STING pathway of APCs and prompting the release of positive immunomodulators. b) Adhesion of NK cells to Pt@PL‐IgG, Pt@L‐IgG, PL‐IgG, Pt@PL, free‐IgG, and PBS treated 4T1 cells by CLSM. NK cells are circled by dashed lines. Scale bar, 20 µm. c, d) Representative flow cytometry plots (c) and quantification (d) of the population of CD69^+^ NK cells after co‐incubation with the different treated 4T1 cells (*n* = 5). e) In vitro cytotoxicity of NK cells against 4T1‐Luc cells with different treatments (*n* = 3). f, g) ATP (f) and LDH (g) levels in the supernatant of 4T1 cells with different treatments followed by co‐incubated with NK cells (*n* = 3). h) Relative gene expression of the cGAS‐STING axis of DC 2.4 co‐incubated with supernatants of 4T1 cells that were differently treated by RT‐qPCR analysis (*n* = 3). i) Representative flow cytometric analysis of the percentage of matured DCs induced by supernatants of 4T1 cells with different treatments. j) Levels of IFN‐1*β* in supernatants of DCs induced by supernatants of 4T1 cells that were differently treated (*n* = 3). Data are shown as mean ± SD; *n* represents the number of biologically independent samples. Student's *t*‐test. *P < 0.05, **P < 0.01 and ***P < 0.001 and ****P < 0.0001.

The ITHAP strategy achieved stable and controlled expression of the tumor cell surface antigen, IgG, which activates NK cells (**Figure** **2**a). First, we investigated whether the illuminated tumor cells could be recognized by NK cells. NK cell adhesion was not observed in the PBS group, suggesting that tumor cells without therapeutic intervention could escape NK cell recognition and achieve malignant proliferation (Figure 2b). The tumor cells illuminated by Pt@PL‐IgG adhered to a large number of NK cells. The number of adherent NK cells in the Pt@PL‐IgG and PL‐IgG groups was similar and was significantly higher than that in the Pt@L‐IgG group, indicating that the platelet membrane enhances the function of bio‐liposomes.

Based on the ability of NK cells to recognize illuminated tumor cells, we next investigated whether NK cells could be activated. The PBS group showed the lowest population of CD69^+^ NK cells, indicating that tumors with poor immunogenicity could not activate NK cells (Figure 2c,d). Compared to the PBS group, the proportion of activated CD69^+^ NK cells in the Pt@PL‐IgG group was significantly increased to 37.9%. Consistent with the NK cell adhesion results (Figure 2b), the abundance of activated NK cells in the Pt@PL‐IgG and PL‐IgG groups was similar and significantly better than that of Pt@L‐IgG, indicating that platelets are essential for the design of bioliposomes.

Next, we investigated the ability of the activated NK cells to induce ADCC. We used luciferase‐labeled tumor cells (4T1‐luc) and a luciferase reporter assay to assess the cytotoxic effects of activated NK cells on tumor cells.^[^ ^16^ ^]^ Bioliposomes could not kill tumor cells without the participation of NK cells (Figure 2e), and the cytotoxicity of the Pt@PL‐IgG group increased to 47% with the participation of NK cells. Further, the apoptosis assay (Figure S8, Supporting Information) demonstrated that NK cells could induce apoptosis in ≈37.5% of illuminated tumor cells. Recent studies have shown that NK cells induce pyroptosis in several tumors.^[^ ^4^ ^]^ Furthermore, we investigated whether NK cells could induce illuminated tumor cell pyroptosis by measuring the release of ATP and lactate dehydrogenase (LDH), which are cytotoxic indicators of pyroptosis.^[^ ^17^ ^]^ We found large amounts of tumor cell‐secreted ATP in the culture medium in the Pt@PL‐IgG group (Figure 2f,g). Moreover, Pt@PL‐IgG treatment resulted in the release of LDH in media from 5.58% (PBS) to 76.0%, further indicating the effectiveness of the ITHAP strategy.

Furthermore, we investigated the activation of the cGAS‐STING pathway in DCs by real‐time quantitative PCR (RT‐qPCR). First, different preparations were incubated with tumor cells for 4 h, then NK cells were added and cultured for 12 h, and then the supernatant was removed and added to DCs for further culturing for 12 h. As shown in Figure 2h, compared with the PBS group, the expression of cGAS‐STING axis genes in DCs of the Pt@PL‐IgG group was significantly increased, indicating activation of the STING signaling pathway, which implies comprehensive activation of the immune response by the bio‐liposomes.

Activation of STING signaling in DCs contributes to DC maturation.^[^ ^18^ ^]^ Therefore, we determined the ratio of mature DCs after different treatments (Figure 2i and S9, Supporting Information). The Pt@PL‐IgG group displayed almost 44.5% mature DCs, compared to 2.69% in the PBS group. Moreover, the Pt@PL‐IgG group further induced the release of large amounts of interferon from DCs (Figure 2j).

### 2.4 In Vivo Biodistribution

In vivo imaging of Pt@PL‐IgG was performed by incorporating the fluorescent agent IR780 into homologous 4T1 cancer‐bearing mice (Figure S10a, Supporting Information). Pt@L‐IgG and Pt@PL‐IgG were injected into mice through the caudal vein to evaluate tumor accumulation. After intravenous injection of Pt@PL‐IgG, the fluorescence intensity at the tumor site accumulated over time and peaked at 24 h, and persisted at the tumor site until 48 h. More importantly, the tumor accumulation in the Pt@PL‐IgG group was significantly higher than that in the Pt@L‐IgG group. Moreover, the isolated organ images also show that Pt@PL‐IgG had excellent tumor accumulation (Figure S10b, Supporting Information). Furthermore, the concentration of Pt at the tumor site further demonstrated the tumor‐targeting and accumulation ability of Pt@PL‐IgG (Figure S10c, Supporting Information).

### 2.5 Immunoefficacy of the Bio‐Liposomes In Breast Cancer

**Figure 3:**
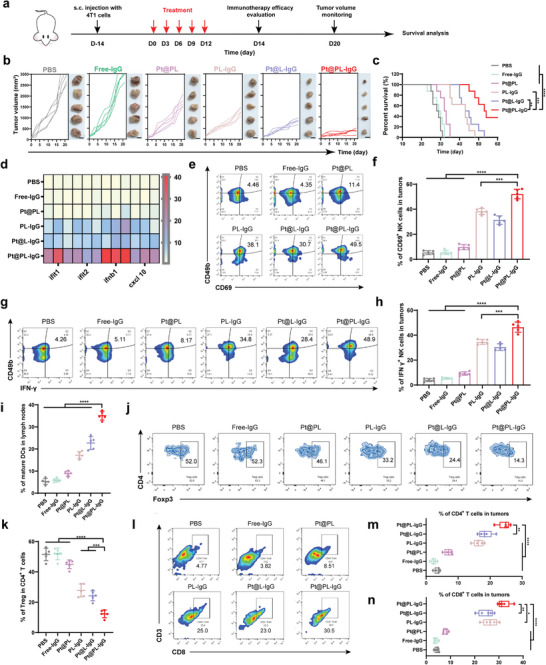
In vivo anti‐tumor efficacy and immune response of ITHAP strategy in 4T1 tumor‐bearing mice. a) Illustration of the experimental protocol in 4T1 tumor‐bearing mice model. b) Image of *ex vivo* 4T1 tumors and individual tumor growth with different treatments (*n* = 5). Scale bar, 1 cm. c) The survival ratio of 4T1 tumor‐bearing mice with different treatments (*n* = 8). d) Relative expression of the cGAS‐STING axis in 4T1 tumors‐bearing mice after different treatments by RT‐qPCR (*n* = 3). e, f) Representative flow cytometry plots (e) and quantification (f) of the population of CD69^+^ NK cells in tumors (*n* = 5). g, h) Representative flow cytometry plots (g) and quantification (h) of the population of IFN *γ* ^+^ NK cells in tumors (*n* = 5). i) Flow cytometry analysis of the population of matured DCs (CD80^+^ and CD86^+^) in the lymph nodes (*n* = 5). j, k) Representative flow cytometry plots (j) and quantification (k) of the population of Tregs (CD4^+^ and Foxp3^+^) in tumors (*n* = 5). l) Representative flow cytometry plots of the effector T cells (CD8^+^ T cells) in tumors. m, n) Flow cytometry analysis of the population of the effector T cells including CD4^+^ T cells (m) and CD8^+^ T cells (*n*) in tumors (*n* = 5). Data are shown as mean ± SD; *n* represents the number of biologically independent samples. Student's *t*‐test. *P < 0.05, **P < 0.01 and ***P < 0.001 and ****P < 0.0001.

We further investigated the immune efficacy of the bio‐liposomes in vivo using a breast cancer model to evaluate their efficacy (**Figure** **3**a). Compared with the PBS group, the PL‐IgG group significantly inhibited tumor proliferation, indicating that the ITHAP strategy is highly efficient and superior (Figure 3b). Notably, the Pt@PL‐IgG group had the strongest curative effectiveness at inhibiting tumor proliferation, whereas treatment with Pt@L‐IgG resulted in moderate tumor inhibition, demonstrating that the platelet membrane is essential for the functioning of bio‐liposomes. HE staining of the tumor site further confirmed the remarkable therapeutic efficacy of Pt@PL‐IgG (Figure S11, Supporting Information). It is also promising to note that Pt@PL‐IgG significantly increased the survival time of mice (Figure 3c). Meanwhile, there was no remarkable change in the body weight of mice treated with the different preparations (Figure S12, Supporting Information).

To validate the successful immune efficacy of the bio‐liposomes, STING signaling pathway activation, and immune cell populations were evaluated. Comparison of the expression of STING‐mediated genes between the Pt@PL‐IgG and PBS groups demonstrated that bio‐liposomes could effectively activate the STING signaling pathway (Figure 3d and Figure S13, Supporting Information).

Moreover, we verified the immune response efficacy of bio‐liposomes by evaluating the populations of NK cells, mature DCs, regulatory T cells (Tregs), and cytotoxic T cells. As one of the most important innate immune cells, NK cells can not only directly kill tumor cells but also secrete positive immunoregulatory factors to activate other immune cells.^[^ ^3^, ^5^ ^]^ Pt@PL‐IgG treatment increased CD69^+^ NK cell infiltration in the tumor from 4.46% (PBS) to 49.5% (Figure 3e,f). Correspondingly, the percentage of CD69^+^ NK cells was ≈38.1% and 30.7% in the PL‐IgG and Pt@L‐IgG groups, respectively. Meanwhile, Pt@PL‐IgG treatment increased the IFN‐*γ* NK cell infiltration in the tumor from 4.26% (PBS) to 48.9% (Figure 3g,h). These results indicate that Pt@PL‐IgG successfully activated NK cells. Adequate activation of NK cells at the tumor site is one of the criteria used to prove the effectiveness of bioliposomes.

DCs are crucial APCs in adaptive immunity and cross‐prime effector T cells.^[^ ^19^ ^]^ We evaluated the populations of mature DCs (CD80^+^ and CD86^+^) in lymph nodes and spleen (Figure 3i, Figures S14 and S15, Supporting Information), and observed that Pt@PL‐IgG treatment increased the population of mature DCs from 5.28% (PBS) to 35.1% in lymph nodes. Meanwhile, the trend of the population of mature DCs in the spleen was similar to that in the lymph nodes. Combined with the in vitro results (Figure 2i), bio‐liposomes could elicit the differentiation of NK cells and DCs for an immune response.

Typically, Tregs (CD4^+^, CD25^+^, and Foxp3^+^) are immunosuppressive cells that are not conducive to an anti‐tumor immune response.^[^ ^20^ ^]^ As shown in Figure 3j, k, the population of Treg infiltration was 52.0% (PBS), indicating that breast cancer is a cold tumor with low immunogenicity. Compared to the PBS group, the population of Treg cells was reduced to 14.3% in the Pt@PL‐IgG group. Next, the frequency of cytotoxic T cells was determined using flow cytometry (Figure 3l–n and Figure S16, Supporting Information). The highest populations of infiltrating T cells (CD4^+^T cells and CD8^+^T cells) were observed in the Pt@PL‐IgG group, at ≈24.7% and ≈30.5%, respectively. Meanwhile, Pt@PL‐IgG treatment exhibited 1.31‐fold and 1.51‐fold higher CD4^+^ T cell infiltration in tumors than the Pt@L‐IgG and PL‐IgG groups, respectively. Overall, these data indicate that the bio‐liposomes resulted in comprehensive and superior immune efficacy in breast cancer. In addition, the biosafety of Pt@PL‐IgG was evaluated by intravenous injection into healthy BALB/c mice. As shown in Figure S17, Supporting Information, Pt@PL‐IgG displayed excellent biological safety and did not cause organ damage or affect liver or kidney function.

### 2.6 Immunoefficacy of the Bio‐Liposomes in Disseminated Xenograft Lymphoma

**Figure 4:**
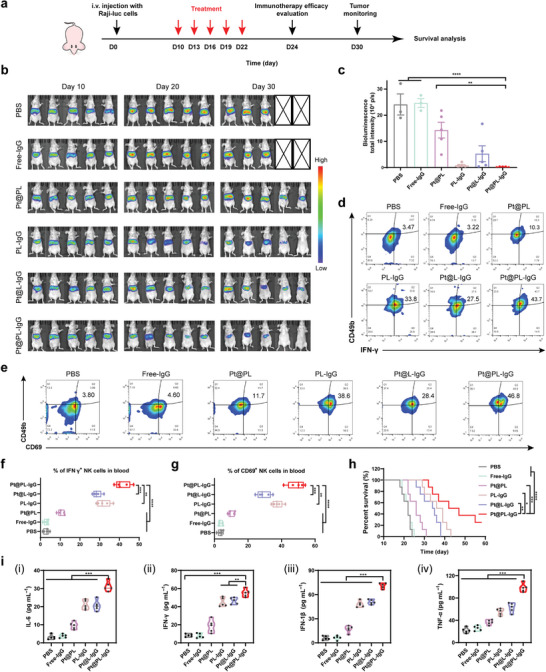
In vivo therapeutic efficacy and activation of the immune response with the ITHAP strategy on disseminated xenograft lymphoma in nude mice. a) Illustration of the experimental protocol in disseminated xenograft lymphoma in nude mice. b,c) In vivo bioluminescence imaging (b) and quantification (day 30) (c) in nude mice with different treatments (*n* = 5). d,f) Representative flow cytometry plots (d) and quantification (f) of the population of IFN‐*γ* ^+^ NK cells in peripheral blood. e, g) Representative flow cytometry plots (e) and quantification (g) of the population of CD69^+^ NK cells in peripheral blood. h) The survival ratio of disseminated lymphoma mice with different treatments (*n* = 8). i) The levels of the serum cytokines (IL‐6, IFN‐*γ*, IFN‐1*β*, and TNF‐*α*) by ELISA (*n* = 5). Data are shown as mean ± SD; *n* represents the number of biologically independent samples. Student's *t*‐test. *P < 0.05, **P < 0.01 and ***P < 0.001 and ****P < 0.0001.

Based on the validation of the efficacy of bioliposomes in breast cancer models, we further evaluated their immune efficacy in lymphoma using a disseminated xenograft lymphoma model (**Figure** **4**a) by assessing lymphoma progression after administration of different preparations using luciferase imaging (Figure 4b,c). Combined with the quantitative analysis data, the lymphoma bioluminescence intensity of the Pt@PL‐IgG group was lower than that of all other groups. We further clarified the underlying immunological mechanisms in the lymphoma model using flow cytometry and ELISA. As shown in Figure 4d–g, after Pt@PL‐IgG treatment, the infiltration of IFN‐*γ* ^+^ NK cells and CD69^+^ NK cells in peripheral blood was 43.7% and 46.8%, respectively. Meanwhile, Pt@PL‐IgG significantly prolonged survival rates in the malignant lymphoma model (Figure 4h). Moreover, there was no remarkable change in the body weight of mice treated with the different preparations (Figure S18, Supporting Information).

This lymphoma model has been established in athymic nude mice, which have obvious T‐lymphocyte immunodeficiency.^[^ ^21^ ^]^ Based on this, we concluded that the anti‐tumor immune efficacy of Pt@PL‐IgG was mainly caused by NK cells. Moreover, it is worth noting that the highest level of interleukin‐6 (IL‐6), IFN‐*γ*, IFN‐1*β*, and tumor necrosis factor‐*α* (TNF‐*α*) were observed in the Pt@PL‐IgG group (Figure 4i). Collectively, the above results demonstrate that the ITHAP strategy, which enhanced the immune recognition of tumor cells, had excellent performance in promoting the activation of NK cells and the immune system.

### 2.7 Immunoefficacy of the Bio‐Liposomes In Pancreatic Cancer

**Figure 5:**
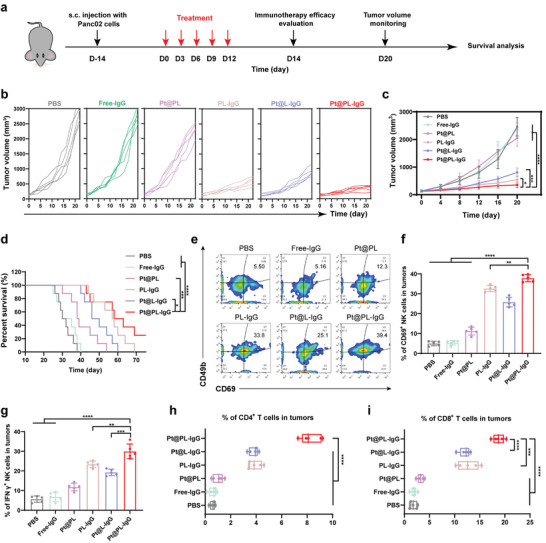
In vivo therapeutic efficacy and activation of the immune response with the ITHAP strategy on Panc02 tumor‐bearing mice. a) Illustration of the experimental protocol in Panc02 tumor‐bearing mice model. b,c) The Panc02 tumors growth curves of mice with different treatments (*n* = 5). d) The survival ratio of Panc02 tumor‐bearing mice with different treatments (*n* = 8). e,f) Representative flow cytometry plots (e) and quantification (f) of the population of CD69^+^ NK cells in tumors (*n* = 5). g) Flow cytometry analysis of the population of IFN‐*γ* ^+^ NK cells in tumors (*n* = 5). h) Flow cytometry analysis of the population of CD4^+^ T cells in tumors (*n* = 5). i) Flow cytometry analysis of the population of CD8^+^ T cells in tumors (*n* = 5). Data are shown as mean ± SD; *n* represents the number of biologically independent samples. Student's *t*‐test. *P < 0.05, **P < 0.01 and ***P < 0.001 and ****P < 0.0001.

We further evaluated the therapeutic efficacy of the ITHAP strategy in pancreatic cancer (**Figure** **5**a). Pancreatic cancer‐bearing mice received various treatments, and we observed that the tumor suppression rate of the Pt@PL‐IgG group was the highest (Figure 5b, c, and Figure S19, Supporting Information). Moreover, there was no remarkable change in the body weight of mice treated with the different preparations (Figure S20, Supporting Information). Notably, treatment with Pt@L‐IgG and PL‐IgG resulted in moderate tumor inhibition, demonstrating that the platelet membrane and Pt are essential for the functioning of the ITHAP strategy. More importantly, compared to the PBS group, the survival time of the Pt@PL‐IgG group was significantly prolonged to 70 days (Figure 5d).

In addition, we verified the immune response efficacy of the ITHAP strategy by evaluating the abundance of CD69^+^ NK, IFN‐*γ* ^+^ NK, CD4^+^ T, and CD8^+^ T cells. As shown in Figure 5e, f, Pt@PL‐IgG treatment increased the CD69^+^ NK cell infiltration in the tumor from 5.50% (PBS) to 39.4%. Correspondingly, the percentage of IFN‐*γ* ^+^ NK cells was ≈29.8% in the Pt@PL‐IgG group. Meanwhile, after Pt@PL‐IgG treatment, the IFN‐*γ* ^+^ NK cell infiltration in the tumor increased from 5.65% (PBS) to 29.8% (Figure 5g and Figure 21, Supporting Information). Of note, after Pt@PL‐IgG treatment, the infiltration of CD4^+^ T cells and CD8^+^ T cells was 8.32% and 18.8%, respectively (Figure 5h,i and Figure S22, Supporting Information). Overall, these data indicate that the ITHAP strategy could promote the activation of NK cells and activate the immune system against pancreatic cancer.

### 2.8 Immunoefficacy of the Bio‐Liposomes in Metastatic Melanoma

**Figure 6:**
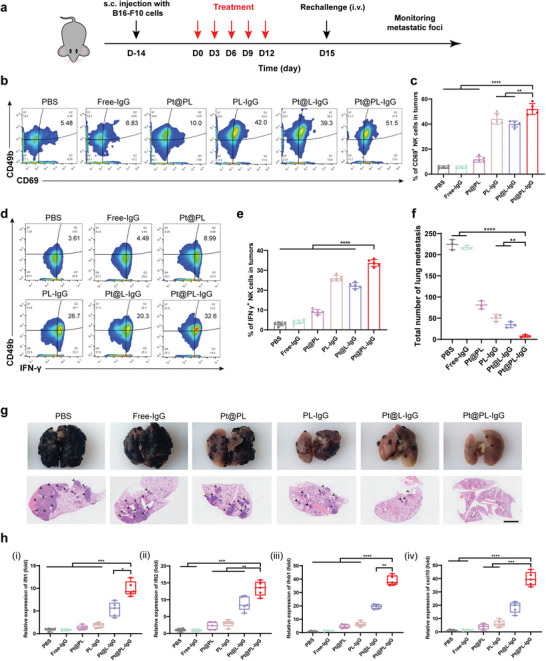
In vivo therapeutic efficacy and activation of the immune response with the ITHAP strategy in metastatic melanoma mice. a) Illustration of the experimental protocol in the metastatic melanoma mice model. b, c) Representative flow cytometry plots (b) and quantification (c) of the population of CD69^+^ NK cells in tumors (*n* = 5). d, e) Representative flow cytometry plots (d) and quantification (e) of the population of IFN *γ* ^+^ NK cells in tumors (*n* = 5). f) Quantitative analysis of the number of metastatic nodules in the lungs on day 30 (*n* = 3). g) Representative images and H&E staining of the lungs after different treatments. Scale bar, 2 mm. h) Relative expression of the cGAS‐STING axis (ifit1, ifit1, ifnb1, and cxcl10) in B16‐F10 tumors‐bearing mice after different treatments by RT‐qPCR (*n* = 5). Data are shown as mean ± SD; *n* represents the number of biologically independent samples. Student's *t*‐test. *P < 0.05, **P < 0.01 and ***P < 0.001 and ****P < 0.0001.

Based on the remarkable immune efficacy of the ITHAP strategy in breast cancer, disseminated xenograft lymphoma, and pancreatic cancer, we further evaluated its therapeutic efficacy in metastatic melanoma (**Figure** **6**a). Compared with the other groups, Pt@PL‐IgG showed the strongest and most significant tumor inhibition effect (Figure S23, Supporting Information). Moreover, there was no remarkable change in the body weight of mice treated with the different preparations (Figure S24, Supporting Information). The abundance of CD69^+^ and IFN‐*γ* ^+^ NK cells in melanoma was detected by flow cytometry. As shown in Figure 6b–e, after Pt@PL‐IgG treatment, the infiltration of CD69^+^ NK cells and in IFN‐*γ* ^+^ NK cell peripheral blood was 51.5% and 32.6%, respectively. This result indicated that the ITHAP treatment strategy showed excellent NK cell activation efficacy in the melanoma model and improved the efficiency of the immune response.

In addition, after treatment, a large number of tumor cells were injected into mice from the tail vein, and their lung metastatic foci were monitored. As shown in Figure 6f,g, numerous metastases were detected in the lungs of mice in the PBS group, indicating successful establishment of the metastatic melanoma model. Compared with the other groups, the lung metastases of mice in the Pt@PL‐IgG group were the least and the smallest. Moreover, PCR results showed that the cGAS‐STING signaling pathway in the lungs of mice in the Pt@PL‐IgG group was fully activated (Figure 6h). Overall, the above data indicate that the ITHAP strategy resulted in comprehensive immune efficacy and tumor metastasis suppression in metastatic melanoma tumors.

## 3 Conclusion

In summary, we proposed an ITHAP strategy based on bio‐liposomes (Pt@PL‐IgG), which achieved stable implantation of an NK‐activatable target antigen on tumor cells to enhance the immune recognition of tumor cells, thus comprehensively triggering an immune response. Pt@PL‐IgG initiated an anti‐tumor immune response by three different mechanisms: 1) illuminating tumor homogenization antigen properties, 2) self‐activation of NK cells, and 3) the release of substantial amounts of interferon to provide a positive immune microenvironment to further potentiate NK therapy. This ITHAP strategy demonstrated impressive immune efficacy in different tumor models, providing a promising approach to address the challenge of tumor heterogeneity.

## 4 Experimental Section

The full materials and experimental methods can be found in the Supporting Information. All animal experiments were conducted in accordance with the evaluation and approved protocols of the ethical committee of China Pharmaceutical University (2022‐12‐007).

## Conflict of Interest

The authors declare no conflict of interest.

## Supporting information

Supporting Information

## Data Availability

The data that support the findings of this study are available from the corresponding author upon reasonable request.
