# Supplementary material for: A Bio‐Liposome Activating Natural Killer Cell by Illuminating Tumor Homogenization Antigen Properties
Source: Adv Sci (Weinh). 2023 Feb 28;10(12):2205449. doi: 10.1002/advs.202205449 (PMC10131854; doi:10.1002/advs.202205449)
Supplement: Supplementary file 1 — Supporting Information [file ADVS-10-2205449-s001.pdf]

ADVANCED SCIENCE
Open Access

Supporting Information

for *Adv. Sci.*, DOI 10.1002/advs.202205449

A Bio-Liposome Activating Natural Killer Cell by Illuminating Tumor Homogenization Antigen Properties

Xue Yang, Jiayi Bian, Zheng Wang, Mengning He, Ying Yang, Quanhao Li, Xinping Luo, Zhanwei Zhou, Jing Li, Shenghong Ju and Minjie Sun**

Supporting Information

A bio-liposome activating natural killer cell by illuminating tumor homogenization antigen properties

Xue Yang[#], Jiayi Bian[#], Zheng Wang, Mengning He, Ying Yang, Quanhao Li, Xinping Luo, Zhanwei Zhou, Jing Li, Shenghong Ju, and Minjie Sun**

J. Bian, Z. Wang, M. He, Y. Yang, Q. Li, L. Luo, Z. Zhou, J. Li, M. Sun
State Key Laboratory of Natural Medicines, Department of Pharmaceutics
Pharmaceutical University
Nanjing, 210009, China
Email: msun@cpu.edu.cn

X. Yang, S. Ju
Department of Radiology, Jiangsu Key Laboratory of Molecular and Functional Imaging
Zhongda Hospital
Medical School of Southeast University
Nanjing, 210009, China
Email: jsh@seu.edu.cn

X. Yang and J.-Y. Bian contributed equally to this work.

Experimental details

Materials

4-(N-Maleimidomethyl) cyclohexane-1-carboxylic acid 3-sulfo-N-hydroxysuccinimide ester sodium salt (sulfo-SMCC sodium salt) was purchased from Aladdin (Shanghai, China). 1,2-distearoyl-sn-glycero-3-phosphoethanolamine-N-[thiol (polyethylene glycol)](DSPE-PEG5000-SH) was purchased from Ponsure Biological (Shanghai, China). Mouse IgG2b Protein (catalog number, 51095-MNAH) and tumor-infiltrating lymphocyte Isolation Kit was obtained from Beijing Solarbio Science & Technology Co., Ltd (Beijing, China). 1,2-Dioleoyl-sn-glycero-3-phosphoethanolamine (DOPE) was purchased from CordenPharma company (Liestal, Switzerland). 1,2-Diacyl-sn-Glycero-3-Phosphocholine (HSPC) was purchased from the AVT (Shanghai) Pharmaceutical Tech Co., Ltd. (Shanghai, China). Cholesteryl hemisuccinate (CHEMS) was purchased from Aladdin (Shanghai, China). Trypsin-EDTA Solution was purchased from T&L Biotechnology Ltd. RPMI-1640 (Beijing, China). MEM, PBS, DiI and 5×SDS-PAGE loading buffer were purchased from KeyGEN BioTECH (Nanjing, China). CELLSAVING (C40100) was bought from New Cell & Molecular Biotech Co.,Ltd (Suzhou, China). Fetal bovine serum (FBS) was bought from ExCell Bio (Shanghai, China). DMEM (Dry powder) was bought from Procell Life Science&Technology Co.,Ltd (Wuhan, China). Anti-CD41and Anti-CD62P Polyclonal antibodies were from Proteintech (Wuhan, China). β-Actin antibody was bought from Bioss (Beijing, China). Anti-rabbit lgG-HRP antibody was bought from Absin (Shanghai, China). DSPE-PEG-FITC was purchased from Tansh-Tech (Guangzhou, China). Hoechst 33258 was purchased from Wuhan Sunncell Biotechnology Co., Ltd (Wuhan, China). cDNA Synthesis Kit and SYBR Green Master Mix (Cat, 11184) were purchased from Yeasen Biotechnology (Shanghai, China). Mouse IFN-1β ELISA Kit and Mouse TNF-α ELISA was purchased from Shanghai Xinyu Biotechnology Co., Ltd (Shanghai, China). Mouse IFN-γ ELISA Kit Kit was purchased from Jiangsu Meibiao Biological Technology Co., Ltd (Jiangsu, China). Mouse IL-6 ELISA Kit was purchased from Lianke Bio (Hangzhou, China). Tumor-infiltrating lymphocyte Isolation Kit was obtained from Beijing Solarbio Science & Technology Co., Ltd (Beijing, China). Glass-bottom dishes and cell culture dishes/plates were purchased from NEST Biotechnology Co. Ltd. (Wuxi, China).

Cell culture and animals

4T1 cells, 4T1-Luc cells, Raji-Luc cells, Panc02 cells and B16-F10 cells were cultured in RPMI-1640 with 10% FBS. DC 2.4 cells were cultured in MEM with 10% FBS. All cells were cultured in a 37°C incubator containing 5% CO_2. Balb/c mice (female, 18-22 g), C57BL/6 mice (male, 18-22 g) and nude mice (female, 18-22 g) were supplied by the Experimental Animal Centre of Yangzhou University (Yangzhou, China). All animal experiments were treated in accordance with the evaluation and approved protocols of the ethical committee of China Pharmaceutical University.

Fabrication and characterizations of Pt@PL-IgG

Synthesis of c,c,t-[Pt(NH_3)$_2$$Cl_2$($O_2CCH_2CH_2CO_2H$)$_2$] (Pt(IV)-COOH) c,c,t-[Pt(NH_3)$_2$$Cl_2$($O_2CCH_2CH_2CO_2H$)$_2$] (Pt(IV)-COOH) was prepared according to the previous reports.[1] Briefly, drop 30% H_2O_2 (50 mL) into the cisplatin aqueous suspension (1 g, 3.33 mmol) in water (30 ml) at room temperature for 4 hours. The initial product c,c,t-[Pt(NH_3)$_2$$Cl_2(OH)_2$] (Pt(IV)) was obtained by filtration and recrystallization. Then stir succinic anhydride (0.9 g, 9 mmol) and Pt(IV) (0.7 g, 2.09 mmol) in 10 mL DMF at room temperature for 24 h. The product Pt(IV)-COOH was precipitated in ether repetitively and then resuspended in water and freeze-dried. The structure of Pt(IV)-COOH was confirmed by ^{1}H NMR.

Synthesis of DSPE-IgG2b conjugate The sulfo-SMCC sodium salt (0.39 mg, 900 nmol) dissolved in PBS(195 μL) was added to the IgG2b (200 μg, 5.4 nmol) solution dissolved in PBS (200 μL). The mixture was shaken at room temperature for 2 hours, and the pH of the whole process was controlled at 7.4. Then, the DSPE-PEG5000-SH(4.5 mg, 900 nmol) solution dissolved in PBS (900 μL) was dropped into the above solution, and the reaction was shaken at room temperature for 4 hours to obtain DSPE-IgG2b.

Extraction of platelet membrane Firstly, add one-tenth of the volume of 4% sodium citrate solution to the mouse whole blood to anticoagulant. Whole blood was then centrifuged at 300 g for 15 min to obtain platelet-containing plasma, which was centrifuged at 800 g for 10 min to harvest mouse platelets. Mouse platelets were separately resuspended in PBS and frozen at -80°C. Then thaw the platelets at room temperature and centrifuge at 10000 g for 5 minutes to pellet. After repeating the above freeze-thaw cycle several times, mouse platelets membrane was obtained. Human platelets were derived from platelet-rich plasma (StemEry, China). And human platelet membrane extraction is the same as that of the mouse.

Preparation and characterizations of Pt@PL-IgG The film dispersion method was used to prepare membrane fusogenic liposomes composed of DOPE/HSPC/CHEMS with a molar ratio of 5.25: 2.49: 1. The detailed process was that DOPE (25 mg), HSPC (12.5 mg) and CHEMS (3.12 mg) were dissolved in dichloromethane and then dried to form a film. Afterward, the film was hydrated with 5 mL PBS containing Pt(IV)-COOH at 37°C for 30 min, and then sonicated with the homogenizer (Biosafer, China) for 30min to obtain liposomes. To prepare Pt@PL-IgG, liposomes were mixed with platelet membranes (20 μg/mg of platelet membrane protein in the total lipid) and the appropriate amount of IgG2b-DSPE and then sonicated for 30 min. The unencapsulated Pt(IV)-COOH and IgG2b-DSPE were removed by centrifugation at 4000 rpm for 20 min at 4°C using ultrafiltration tubes (MWCO 50K, Millipore, USA). To verify the stability of Pt@PL-IgG, it was incubated in 1640 medium + 10% FBS and PBS buffer for 60 h. The particle size, polydispersity index (PDI) and zeta potential of Pt@PL-IgG were measured with a DLS spectrophotometer (Malvern, England). Concurrently, the transmission electron microscopy (TEM) imaging confirmed the morphology of Pt@PL-IgG and Pt@L-IgG.

Preservation of platelet membrane on Pt@PL-IgG

The preservation of the platelet membrane on Pt@PL-IgG was verified by Western blot analysis. Firstly, the BCA assay kit (Beyotime, China) was used to quantify the total protein concentration of platelets, platelet membranes and Pt@PL-IgG. Then pipette SDS-PAGE sample loading buffer into these samples, and heat them at 100°C. The related proteins were separated by SDS-polyacrylamide gel electrophoresis (SDS-PAGE) and transferred by PVDF membrane (Millipore, USA). The PVDF membrane was blocked with 5% skimmed milk and incubated with anti-CD41, anti-CD62P, and β-Actin antibodies at 4°C overnight. After incubating with the secondary antibody, the membrane was imaged with ECL immunoblotting substrate and ECL imager (Tanon, China). In addition, the above samples along with the IgG2b protein were also stained with a coomassie brilliant blue solution to observe the protein distribution.

***In vitro* cellular uptake and the mechanisms of uptake**

Rhodamine B was encapsulated in liposomes to make Rhb@PL-IgG containing platelet membrane and Rhb@L-IgG without platelet membrane. 4T1 cells were seeded in the 24-well plate and confocal dishes and grown at 37°C for 24 hours. Rhb@PL-IgG, Rhb@L-IgG, and PBS were added to 4T1 cells and incubated for 3 h, 2 h and 1 h. Then detect the intracellular

fluorescence intensity of rhodamine B by confocal laser scanning microscopy (CLSM) and flow cytometer (BD FASC Verse).

After 4T1 cells were seeded in a 24-well plate for 24 hours, PBS, chlorpromazine (inhibition of clathrin-mediated endocytosis), cytochalasin D (inhibition of phagocytosis), wortmannin (inhibition of macropinocytosis) and PBS at 4°C were added respectively to the cells and incubated for 30 minutes. Then add Rhb@PL-IgG to cells and incubate for 2 hours. Finally, the intracellular fluorescence intensity was detected by flow cytometry.

Membrane fusogenic capability of liposomes

DSPE-PEG-FITC (M.W.1000) was incorporated into liposomes to confirm the capability of membrane fusion. Liposomes composed of DOPE/HSPC/DSPE-PEG-FITC/CHEMS (molar ratio of 5.25: 2.49: 1.6:1) were prepared and divided into two groups platelet membrane added (Pt@PL-FITC) and without platelet membrane (Pt@L-FITC). 4T1 cells were seeded in confocal dishes for 24 h, and then Pt@PL-FITC and Pt@L-FITC were added and incubated for 2 h, 4 h, 12 h and 24 h. The dye DiI of the membrane and the dye Hoechst of the nucleus were used to stain the cells. Finally, observe the fluorescence distribution of the cells by CLSM (Olympus FV300).

Verification of IgG retention on the cell membrane

Goat anti-Mouse IgG-FITC (Solarbio, China) was allowed to replace IgG2b as a fluorescently labeled IgG to verify that Pt@PL-IgG leaves IgG on the cell membrane. IgG-FITC (0.3 ml) and sulfo-SMCC (0.15 mg, dissolved in 37.5 µl PBS) at a molar ratio of 1:20 were oscillated for 2 h at room temperature to synthesize SMCC-IgG-FITC. DSPE-PEG-SH (1.8 mg, dissolved in 120 µl PBS) was then added to SMCC-IgG-FITC and shaken for 4 h at room temperature to obtain DSPE-IgG-FITC. DSPE-IgG-FITC was mixed with membrane fusion liposomes and sonicated by the homogenizer to compound Pt@PL-IgG, Pt@L-IgG and PL-IgG.

4T1 cells were inoculated in confocal dishes for 24 h at 37°C and then incubated for 12 h with Pt@PL-IgG, Pt@L-IgG, PL-IgG, Pt@PL, free-IgG and PBS (IgG2b was 2 ug/ml and Pt(IV)-COOH was 10 ug/ml in each group). IgG expression on cells was detected by flow cytometry.

In vitro isolation of mouse NK cells and the adhesion of NK cells to tumor cells

Mouse NK cells were obtained from the mouse spleen. The mouse spleen NK cell isolation kit (TBD science, China) was used to conduct preliminary isolation of NK cells. Then BD IMag™ Mouse NK Cell Separation Set-DM was applied to further isolate NK cells according to the manufacturer's instructions.

The 4T1 cells seeded in the confocal dish were co-cultured with Pt@PL-IgG, Pt@L-IgG, PL-IgG, Pt@PL, free-IgG and PBS for 4 hours at 37°C. The concentration of IgG2b in each group was 2 ug/ml and that of Pt(IV)-COOH was 10 ug/ml. Then NK cells were added to 4T1 cells in a ratio of 1:1 and co-incubated for 4 hours at 37°C. 4% paraformaldehyde was used to fix the cells and Hoechst was used to stain the nucleus. Fluorescence images were finally recorded by CLSM.

In vitro activation of NK cells

The activation of NK cells was assessed by the expression of CD69 in mouse NK cells. 4T1 cells were cultured in 24-well plates for 24 h at 37°C. After incubation of the cells with Pt@PL-IgG, Pt@L-IgG, PL-IgG, Pt@PL, free-IgG and PBS for 4 h, an equal number of NK cells as tumor cells were added and incubated for 12 h. NK cells were collected by centrifugation of the cell supernatant and stained with anti-CD69-PE-Cy7 (BD). The percentage of $CD69^{+}$ in each group was detected by flow cytometry.

In vitro validation of the ADCC effect

Antibody-dependent cell-mediated cytotoxicity (ADCC) of NK cells was verified by Annexin V-FITC/PI apoptosis assay and bioluminescence of luciferase-expressing cell lines. The first 4T1 cells were inoculated in 24-well plates and incubated for 24 h at 37°C. Then the cells were co-incubated with Pt@PL-IgG, Pt@L-IgG, PL-IgG, Pt@PL, free-IgG and PBS for 4 h and equal numbers of NK cells were added and incubated for 24 h. According to the manufacturer's recommendations, tumor cells were stained for phosphatidylserine (PS) and nuclei by Annexin V-FITC/PI Apoptosis Kit (K2003, APExBIO, Houston, USA) and detected on flow cytometry.

Luciferase transfected cells catalyze luciferin to emit bioluminescence at a wavelength of approximately 560 nm without occurring in dead cells. Luciferase-transfected 4T1 cells were inoculated in 96-well plates and incubated for 24 h at 37°C. The cells were then incubated with Pt@PL-IgG, Pt@L-IgG, PL-IgG, Pt@PL, free-IgG and PBS for 4 h before adding equal numbers of NK cells for 24 h. After processing the tumor cells with One-Lumi™ Firefly

Luciferase Assay Kit (Beyotime, China) according to the manufacturer's recommendations, the cells were assayed for bioluminescence by the Synergy2 enzyme marker (Bio-Tek, USA).

***In vitro* dendritic cells (DCs) maturation studies**

4T1 cells were cultured in 24-well plates and incubated with Pt@PL-IgG, Pt@L-IgG, PL-IgG, Pt@PL, free-IgG, and PBS for 4 h. Equal amounts of NK cells were then added to co-incubate for 12 h. DC 2.4 was inoculated in 24-well plates at 37°C for 24 h. Then the supernatants of the above 4T1 cells were added to DC 2.4 for 24 h. Finally, DCs were stained with anti-CD86-APC (BD) and anti-CD80-PE (BD) antibodies to detect the maturity by flow cytometry.

***In vitro* analysis of cGAS-STING pathway**

Real-time quantitative fluorescence PCR (qPCR) was used to verify the activation of the cGAS-STING pathway in vitro. 4T1 cells were seeded in 12-well plates for 24 h and treated with Pt@PL-IgG, Pt@L-IgG, Pl-IgG, Pt@PL, free-IgG and PBS for 4 hours. Afterward, mouse NK cells were added to 4T1 cells and cultured together for 12 hours. DC 2.4 cells were seeded in a 12-well plate for 24 h. Then the supernatants from 4T1 cells were added to DC 2.4 cells and incubated for 12 hours. The FastPure® Cell/Tissue Total RNA Isolation Kit V2 （Vazyme Biotech Co.,Ltd, Chian） was used to extract RNA from DC 2.4 cells. According to the recommendations of the reagent manufacturer, complementary DNA (cDNA) was obtained by the cDNA synthesis kit. And the cDNA was mixed with SYBR Green Master Mix for qPCR analysis.

***In vitro* verification of pyroptosis**

Pyroptosis was verified by the leakage of ATP and lactate dehydrogenase (LDH) in cells. 4T1 cells were cultured in 96-well plates and incubated with Pt@PL-IgG, Pt@L-IgG, PL-IgG, Pt@PL, free-IgG and PBS for 4 h, followed by incubation with equal numbers of NK cells for 24 h. Leakage of LDH and ATP in the supernatant of drug-treated tumor cells were detected using the LDH Cytotoxicity Assay Kit and ATP Assay Kit (Beyotime, China) according to the manufacturer's recommendations.

***In vitro* analysis of IFN-1β release**

4T1 cells were cultured in 96-well plates and incubated with Pt@PL-IgG, Pt@L-IgG, PL-IgG, Pt@PL, free-IgG and PBS for 4 h, followed by incubation with equal numbers of NK

cells for 12 h. DC 2.4 were inoculated in 24-well plates at 37°C for 24 h. Then the supernatants of the above 4T1 cells were added to DC 2.4 for 24 h. The DC 2.4 cell culture supernatants were then collected and centrifuged. The IFN-1β in the supernatant of each group was determined by ELISA kit.

In vivo biodistribution

IR780 iodide dye (Sigma-Aldrich, USA) was dissolved in ethanol and incorporated into liposomes to prepare Pt@PL-IgG and Pt@L-IgG. 4T1 tumor-bearing female balb/c mice were intravenously injected with Pt@PL-IgG and Pt@L-IgG at a dose of 2 mg/kg IR780 respectively. The fluorescence signals of the whole body and organs of mice were then examined at 2, 6, 12, 24, 36 and 48 h by In Vivo Imaging System (IVIS) (PerkinElmer, USA). Meanwhile, tumors were obtained from mice at 2, 6, 12, 24, 36 and 48 h for determination of the cisplatin content by inductively coupled plasma mass spectrometry (ICP-MS).

In vivo efficacy on 4T1 tumor-bearing mouse model

To construct a 4T1 tumor-bearing mouse model, 1×10^6 4T1 cells were injected into the breast pads of female balb/c mice. When the tumor volume reached 100 mm^3, the mice were divided into 6 groups and Pt@PL-IgG, Pt@L-IgG, Pl-IgG, Pt@PL, free-IgG and PBS (IgG2b at a dose of 100 μg/kg and Pt(IV)-COOH at a dose of 2 mg/kg) were injected into the tail vein on days 0, 3, 6, 9 and 12 respectively. The tumor volume, body weight and survival time of the mice were recorded every other days (tumor volume calculation formula: $V=0.5 \times width^2 \times length$).

On day 14, tumors were obtained and subjected to qPCR analysis of the cGAS-STING pathway and hematoxylin-eosin (H&E) staining analysis. In addition, tumors and lymph nodes of 4T1 tumor-bearing mice were removed to verify the immunization effect. To analyze dendritic cells, digested lymph node cells and spleen cells were stained with anti-CD45-FITC (BD), anti-MHCII-bv421 (BD), anti-CD11c-PE-Cy7 (BD), anti-CD86-APC (BD) and anti-CD80-PE (BD) antibodies. To analyze the activation of NK cells, cells obtained from tumors were first stimulated with Leukocyte Activation Cocktail, with BD GolgiPlug™ (BD) for 4-6 hours and then stained with anti-CD45-FITC (BD), anti-CD3-percp-cy5.5 (BD), anti-CD49b-PE (BD), anti-CD69-PE-Cy7 (BD) and anti-IFN-APC (BD) antibodies. To detect the proportion of $CD8^+$ T cells, $CD4^+$ T cells and Treg cells ($CD4^+$, $CD25^+$ and $Foxp3^+$) in the tumor, lymphocytes in the tumor were stained with anti-CD45-FITC (BD), anti-CD3-PerCP-Cy™5.5 (BD), anti-CD4-APC (BD), anti-CD8-PE-Cy7 (BD), anti-CD25-bv421 (BD) and

anti-Foxp3-PE (BD) antibodies. The stained cells were finally detected by flow cytometry (BD FASC Verse).

In vivo anti-disseminated xenograft lymphoma efficacy

To establish disseminated Raji B-cell xenograft lymphoma, 2×10^6 Raji-luc cells were intravenously injected into the tail vein of female nude mice. 10 days after inoculation, mice were divided into 6 groups and administered intravenously Pt@PL-IgG, Pt@L-IgG, Pl-IgG, Pt@PL, free-IgG and PBS on days 10, 13, 16, 19 and 22. Simultaneously, bioluminescence imaging was performed on days 10, 20 and 30 after the injection of tumor cells by IVIS to monitor the disseminated tumors (10-15 min after injection of 150 mg/kg D-luciferin). The survival time of the mice was recorded. The body weight of mice in each group were recorded every other day.

To detect the activation of NK cells, anticoagulated whole blood was collected from the orbital vein of mice on day 24. Three times the volume of erythrocyte lysate was added to 200 μl of whole blood and the supernatant was discarded by centrifugation. The obtained cells were then analyzed by flow cytometry after the addition of the appropriate stimulants and staining with antibodies as described previously. Meanwhile, the remaining blood samples were centrifuged to obtain serum. Then, the serum was assayed for cytokines, including IL-6, IFN-γ, IFN-1β and TNF-α by ELISA kits according to the manufacturer's method.

In vivo efficacy on Panc02 tumor-bearing mouse model

To construct the Panc02 tumor-bearing mouse model, 1×10^6 panc02 cells were injected subcutaneously into male C57BL/6 mice. When the tumor volume reached 100 mm^3, the mice were divided into 6 groups and Pt@PL-IgG, Pt@L-IgG, Pl-IgG, Pt@PL, free-IgG and PBS were injected into the tail vein on days 0, 3, 6, 9 and 12 respectively. The tumor volume, body weight and survival time of the mice were recorded every other day.

On day 14, tumors were obtained and subjected to H&E staining analysis. In addition, tumors of Panc02 tumor-bearing mice were removed to verify the immunization effect (NK cells, $CD8^+$ T and $CD4^+$ T cells in tumors). The obtained tumor cells were then analyzed by flow cytometry after appropriate stimulation and staining with antibodies as described previously.

In vivo efficacy on metastatic melanoma mouse model

To construct the metastatic melanoma mouse model, 1×10^6 B16-F10 cells were injected subcutaneously into male C57BL/6 mice (primary tumors). When the tumor volume reached 100 mm^3, the mice were divided into 6 groups and Pt@PL-IgG, Pt@L-IgG, Pl-IgG, Pt@PL, free-IgG and PBS were injected into the tail vein on days 0, 3, 6, 9 and 12 respectively. The tumor volume of the mice was recorded every two days. On day 14, tumors were obtained and subjected to qPCR analysis of the cGAS-STING pathway. Meanwhile, the ratio of activated NK cells in tumors was determined by flow cytometry. Next, the remaining mice in each group were intravenously re-injected B16-F10 cells (1×10^5) for tumor rechallenge. On day 30, lungs from each group of mice were collected and fixed in paraformaldehyde. Subsequently, the number of metastatic nodules in the lungs was recorded and H&E staining was performed. The body weight of mice in each group were recorded every other day.

Biological Safety Evaluation of Pt@PL-IgG

The biosafety of Pt@PL-IgG was evaluated in healthy Balb/c mice. Mice in the Pt@PL-IgG group were intravenously injected with Pt@PL-IgG (IgG2b at a dose of 100 μg/kg and Pt(IV)-COOH at a dose of 2 mg/kg) and mice in the control group were intravenously injected with saline. Two weeks later, major organs of mice were harvested for H&E staining analysis. Meanwhile, blood was collected from mice and serum was isolated for the measurement of alanine aminotransferase (ALT), aspartate aminotransferase and urea nitrogen (BUN) levels to evaluate liver and kidney damage. Several hematological tests were also performed on whole blood.

Statistical analysis

All experimental data were performed 3 or more times independently. All statistical analyses were performed with GraphPad Prism 8.0 software. Results were expressed as mean ± SD. To compare statistical differences between the two groups, a two-tailed Student's t-test was performed for statistical analysis. Significant values are shown below: $^*P < 0.05$, $^{**}P < 0.01$, $^{***}P < 0.001$ and $^{****}P < 0.0001$.

Supporting figures

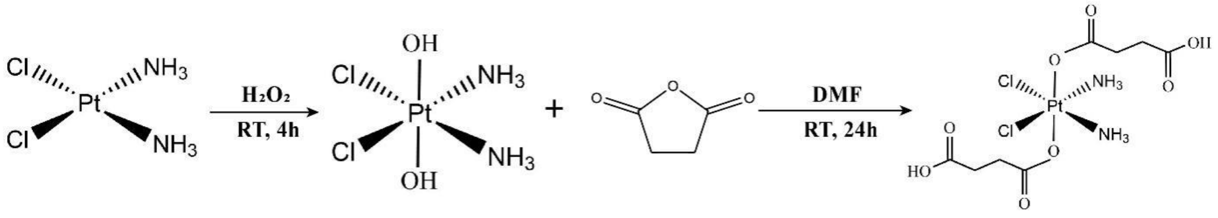

Cisplatin Pt(IV) SA Pt(IV)-COOH

Figure S1. Synthesis of Pt(IV)-COOH.

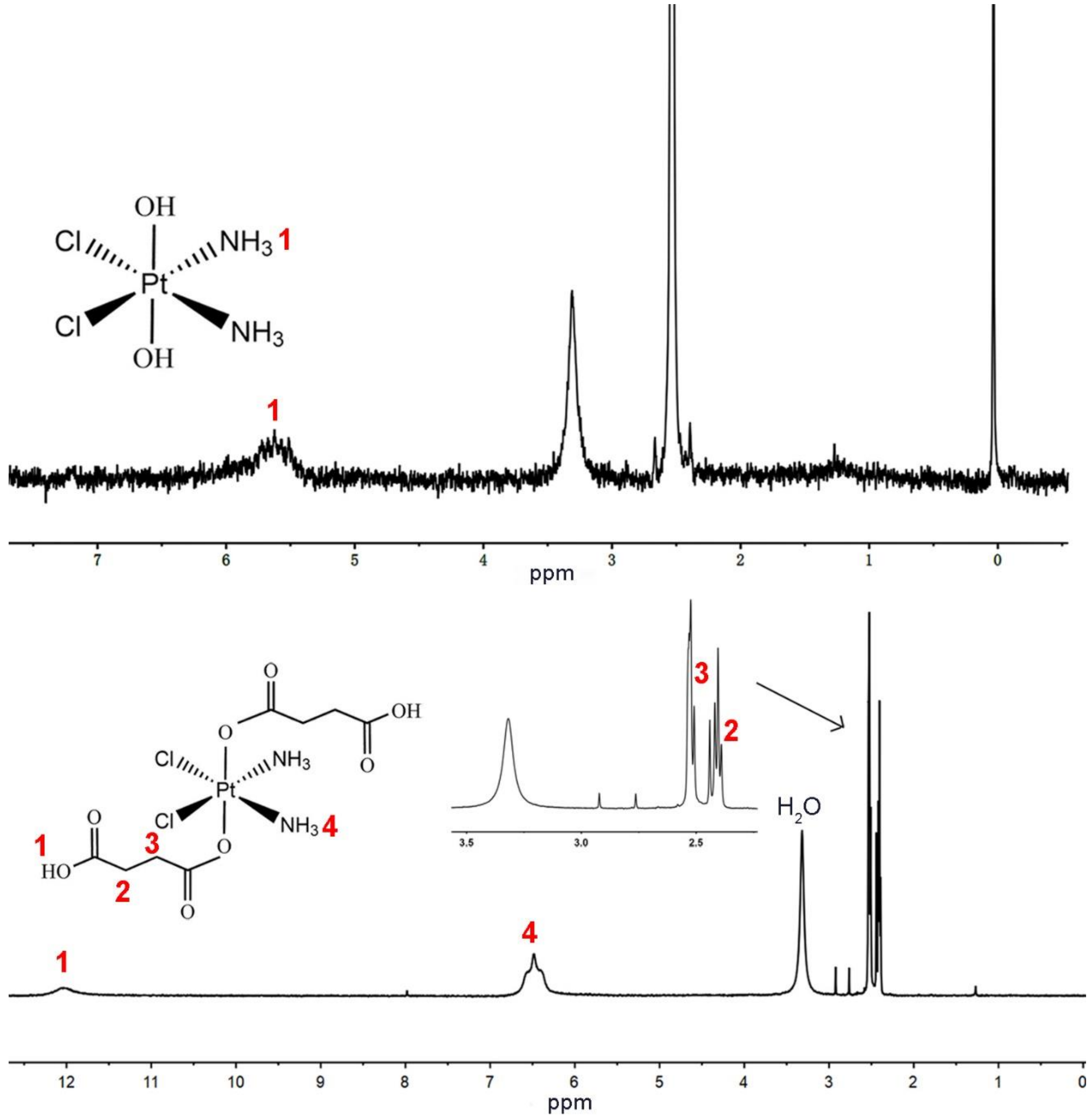

Figure S2. ^{1}H NMR of Pt(IV)-COOH.

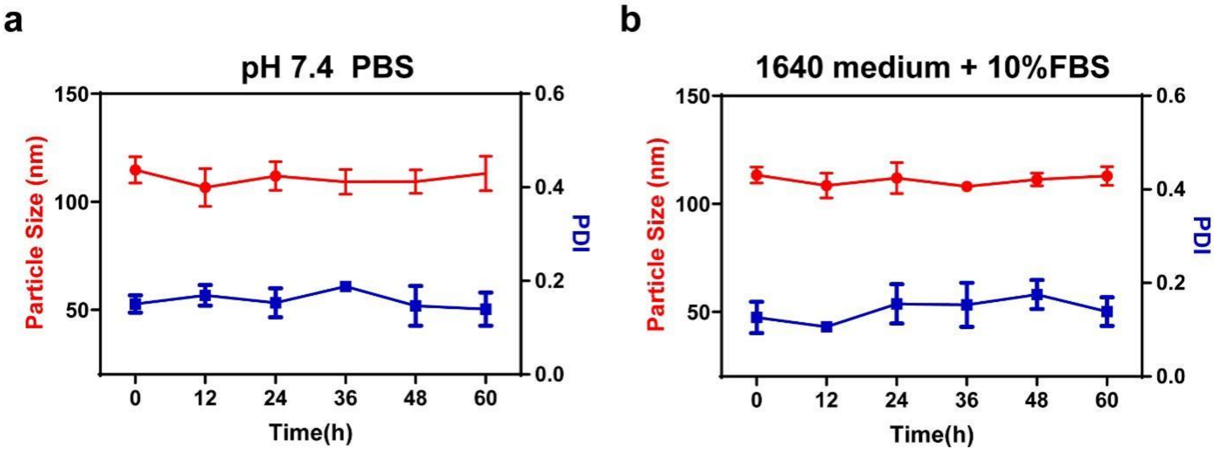

Figure S3. *In vitro* stability validation of Pt@PL-IgG. Size distribution and PDI changes of Pt@PL-IgG in 60h at PBS buffer **(a)** and 1640 medium + 10%FBS **(b)**.

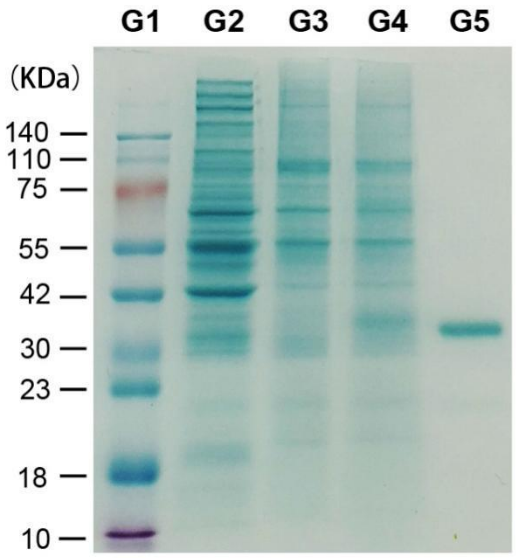

Figure S4. SDS-PAGE protein analysis of (G1) marker, (G2) platelets, (G3) platelet membrane, (G4) Pt@PL-IgG, and (G5) IgG.

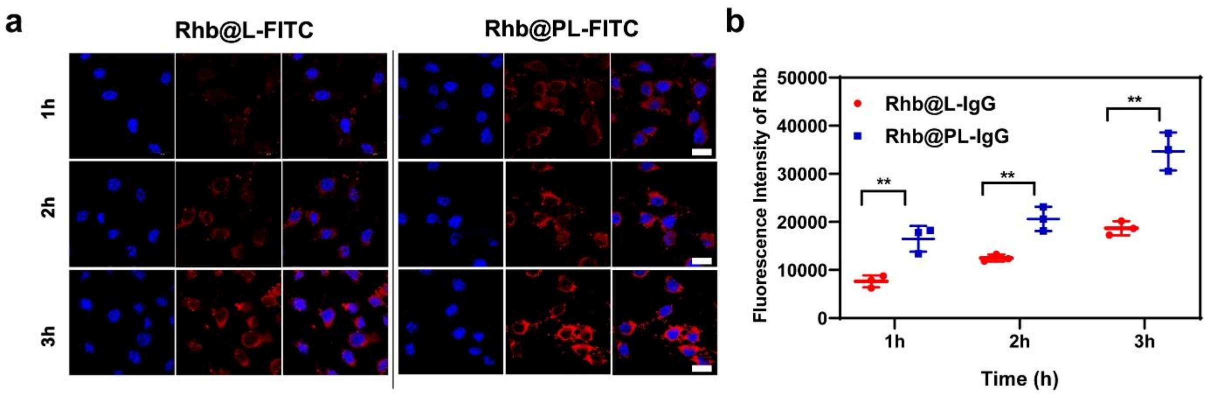

Figure S5. Cellular uptake of bio-liposomes *in vitro*. (**a**) Fluorescent imaging of 4T1 cells incubated with Rhb@L-IgG and Rhb@PL-IgG. Red: rhodamine b, blue: hoechst. Scale bar = 20 μm. (**b**) Cellular uptake of Rhb@L-IgG and Rhb@PL-IgG were analyzed by flow cytometry (n=3). Data are shown as mean ± SD; n represents the number of biologically independent samples. Student's t-test. $^{*}P < 0.05$, $^{**}P < 0.01$ and $^{***}P < 0.001$ and $^{****}P < 0.0001$.

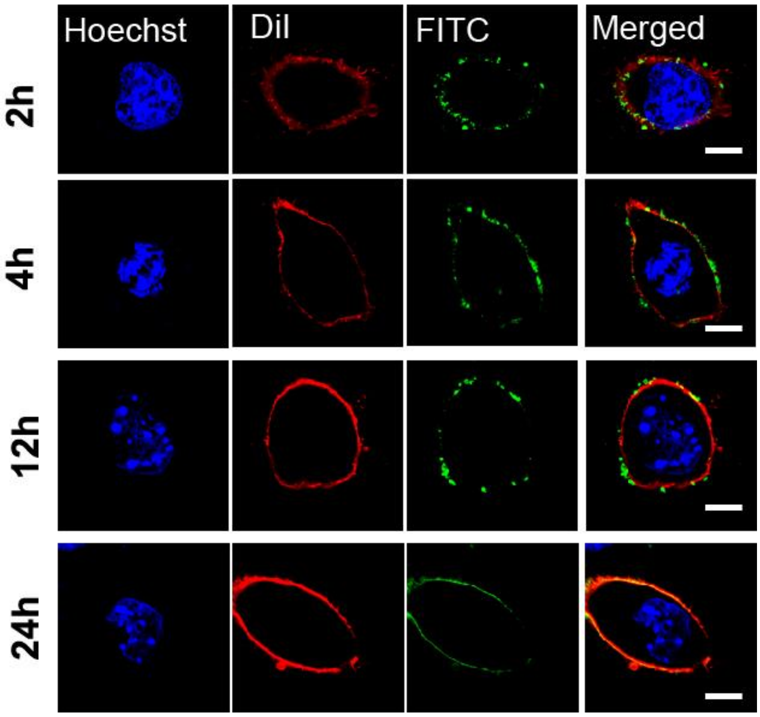

Figure S6. Fusogenic property of Pt@L-FITC by the CLSM. Phospholipids were labeled with FITC (green), tumor cell membrane labeled with DiI (red), and tumor cell nucleus labeled with Hoechst (blue). Scale bar = 5 μm.

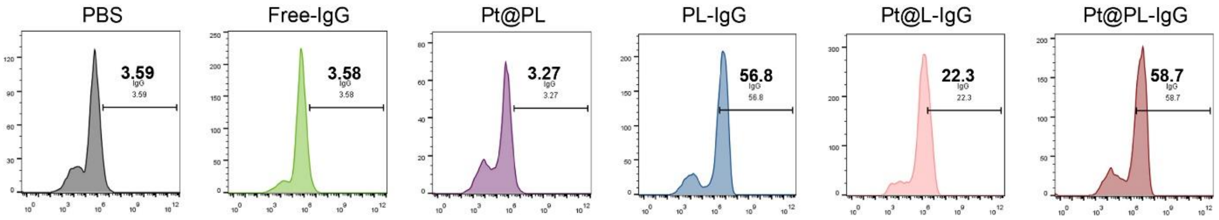

Figure S7. The expression of IgG-FITC in 4T1 cells after different treatments by flow cytometry *in vitro*.

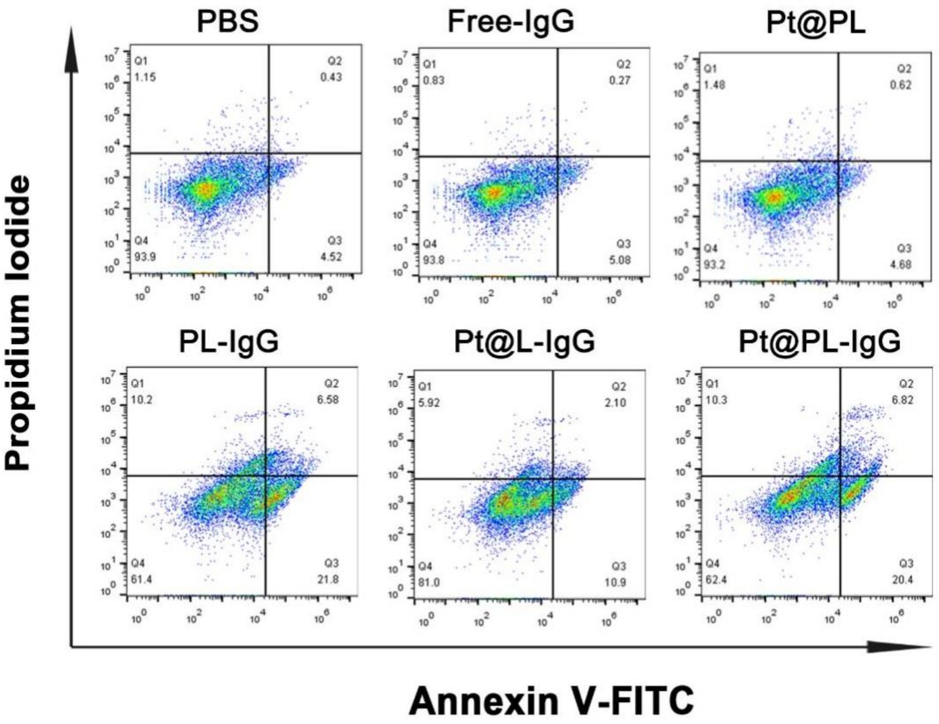

Figure S8. *In vitro* apoptosis analysis of 4T1 cells with different treatment by Annexin V-FITC/PI staining.

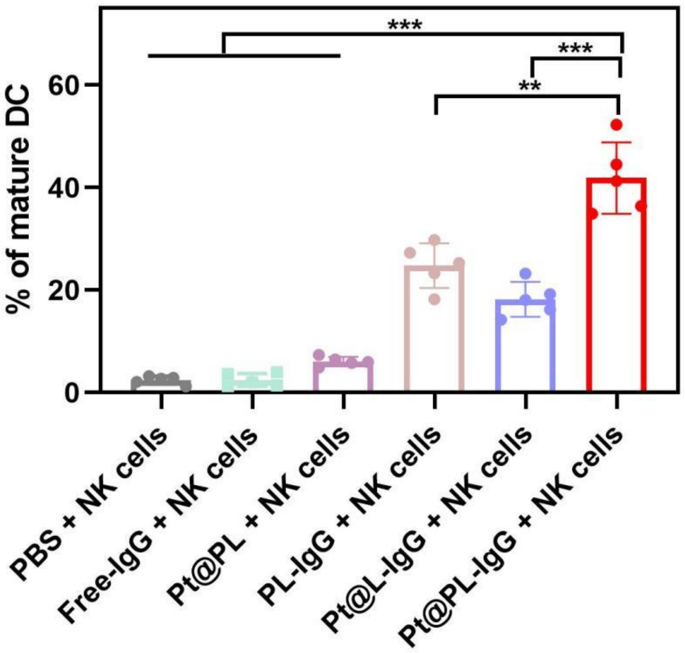

Figure S9. The efficacy of promoting DC maturation evaluated by flow cytometry *in vitro*. Data are shown as means ± SEM (n=5). Data are shown as mean ± SD; *n* represents the number of biologically independent samples. Student's t-test. *P < 0.05, **P < 0.01 and ***P < 0.001 and ****P < 0.0001.

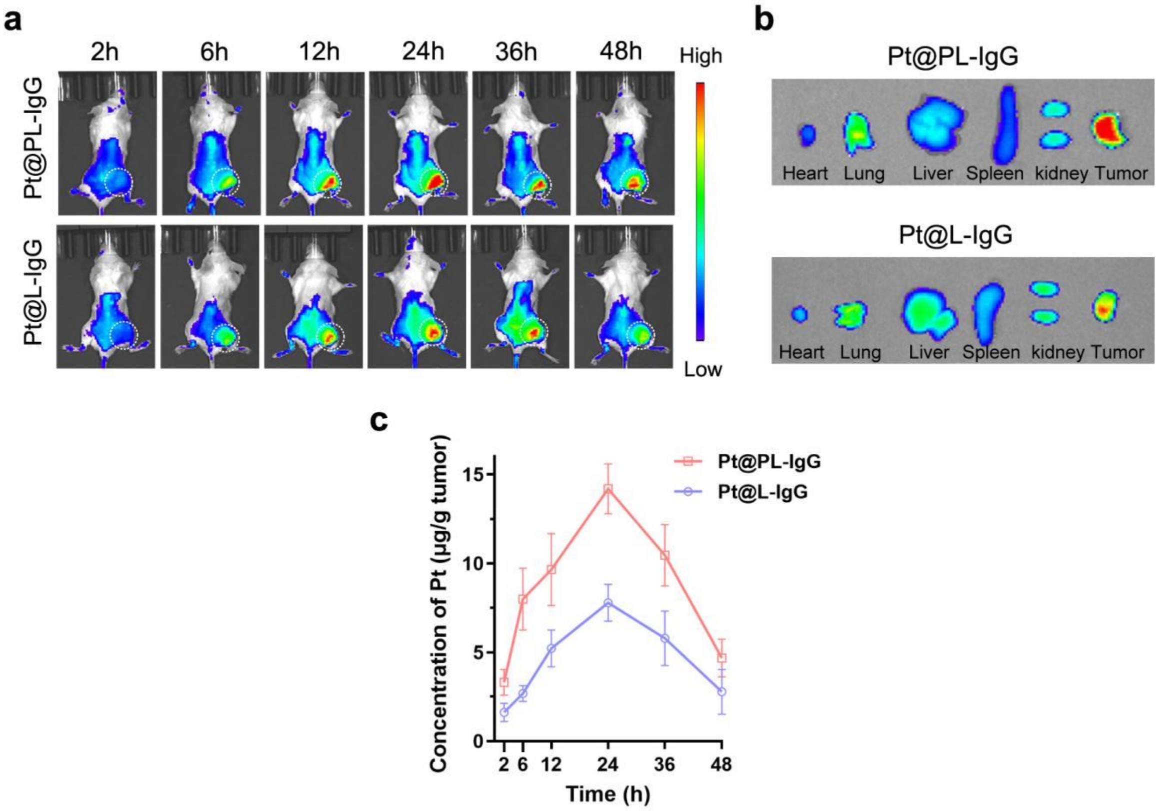

Figure S10. *In vivo* biodistribution of bio-liposomes in 4T1 tumor-bearing mice. (**a**) Fluorescence images of 4T1 tumor-bearing mice after intravenous injection of Pt@PL-IgG and Pt@L-IgG *in vivo*. (**b**) Representative fluorescence images of major organs removed from mice 24 hours after intravenous injection of Pt@PL-IgG and Pt@L-IgG. (**c**) Intratumoral cisplatin content in 4T1 tumor-bearing mice after intravenous injection of Pt@PL-IgG and Pt@L-IgG (n=3).

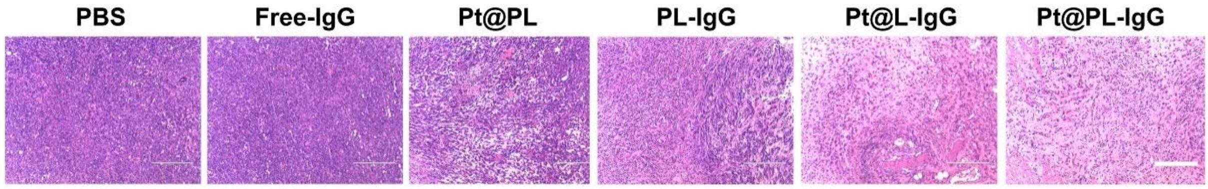

Figure S11 H&E staining of 4T1 tumor tissues with different treatments. Scale bar, 200 μm.

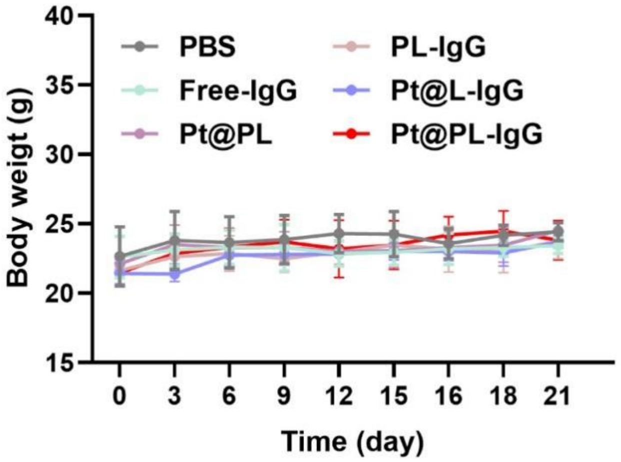

Figure S12. The body weight curves of 4T1 tumor-bearing mice (n=5).

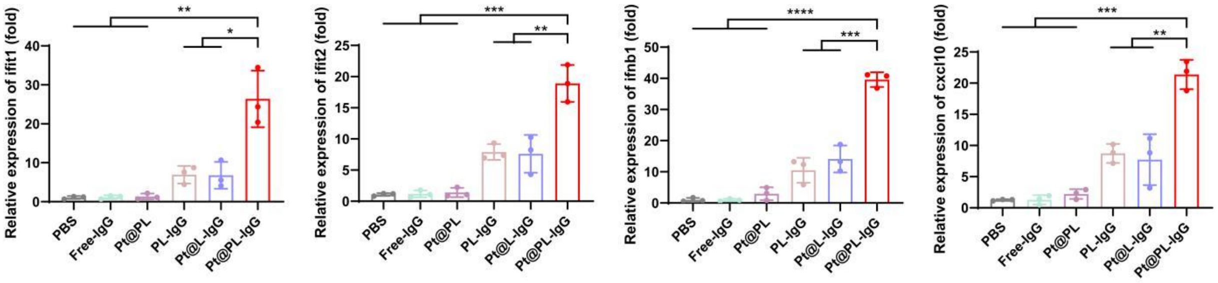

Figure S13. Relative expression of the cGAS-STING axis (ifit1, ifit1, ifnb1, and cxcl10) in B16-F10 tumors-bearing mice after different treatments by RT-qPCR (n=5). Data are shown as mean ± SD; *n* represents the number of biologically independent samples. Student's t-test. *P < 0.05, **P < 0.01 and ***P < 0.001 and ****P < 0.0001.

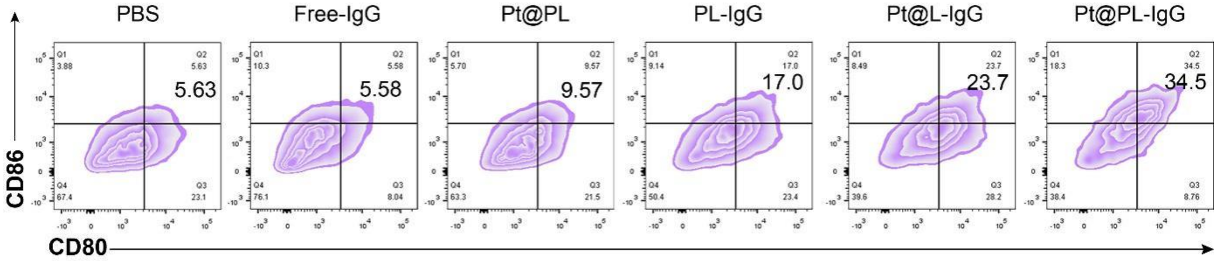

Figure S14. Representative flow cytometric analysis of the percentage of matured DCs (CD80^{+} and CD86^{+}) in the lymph nodes in the 4T1 tumor-bearing mice model.

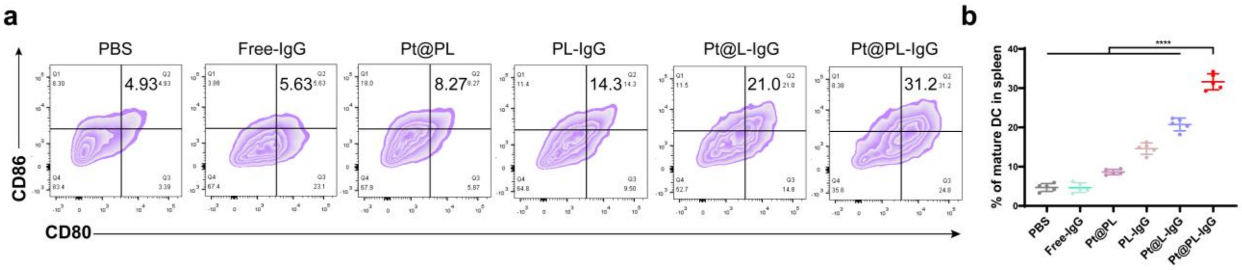

Figure S15. (**a, b)** Representative flow cytometric analysis (a) and quantification (b) of the percentage of matured DCs (CD80$^+$ and CD86$^+$) in the spleen in the 4T1 tumor-bearing mice model.

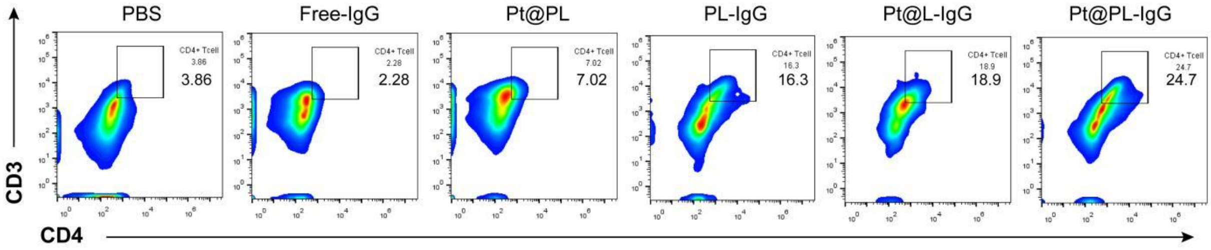

Figure S16. Representative flow cytometric analysis of the percentage of CD4$^+$ T cells in tumors in the 4T1 tumor-bearing mice model.

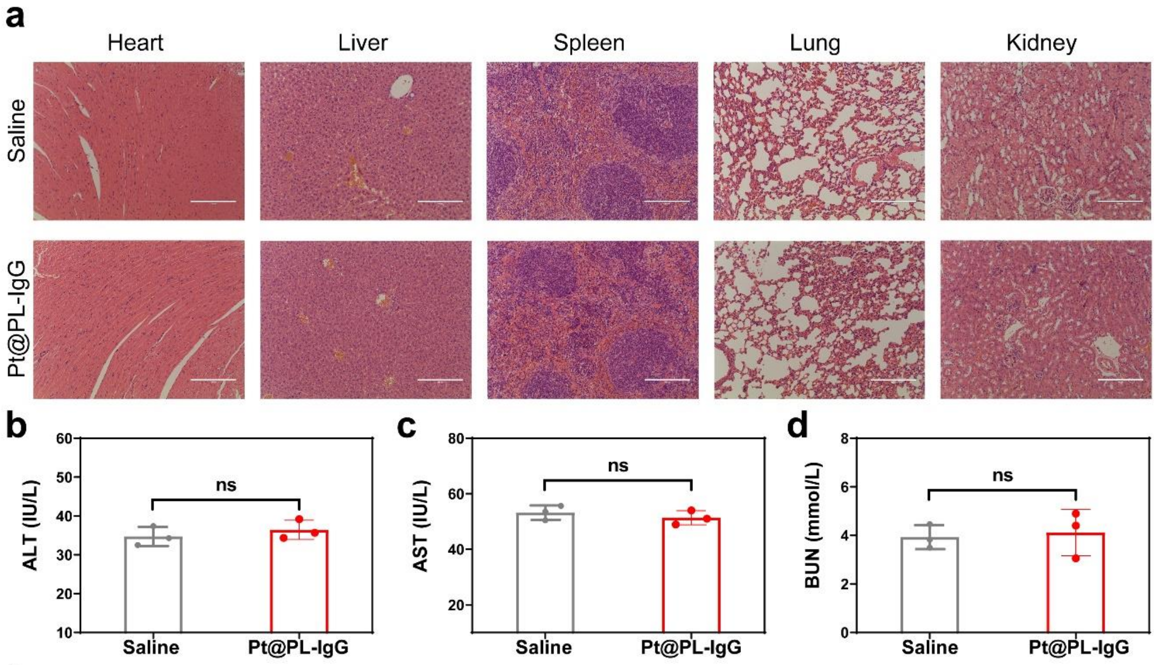

e

Samples	WBC (10^9/L)	RBC (10^{12}/L)	Hemoglobin (g/L)	Platelet (10^9/L)	Neutrophil (10^9/L)	Lymphocyte (10^9/L)	Monocyte (10^9/L)
Saline	6.45±0.23	9.31±0.45	126.3±4.14	578.2±12.2	0.42±0.02	4.98±0.42	0.10±0.01
Pt@PL-IgG	6.21±0.56	9.28±0.52	124.2±3.84	583.6±10.2	0.39±0.04	5.12±0.35	0.11±0.01

Figure S17. (a) H&E stained images of the major organs (heart, liver, spleen, lung and kidney) taken from mice after Pt@PL-IgG treatment. Scale bar, 200 μm. **(b-d)** Serum levels of ALT (b), AST (c) and BUN (d) after Pt@PL-IgG treatment in mice (n=3). **(e)** Hematological parameters after Pt@PL-IgG treatments in mice (n=3). Data are shown as mean ± SD; n represents the number of biologically independent samples. Student's t-test. $^{*}P < 0.05$, $^{**}P < 0.01$ and $^{***}P < 0.001$ and $^{****}P < 0.0001$.

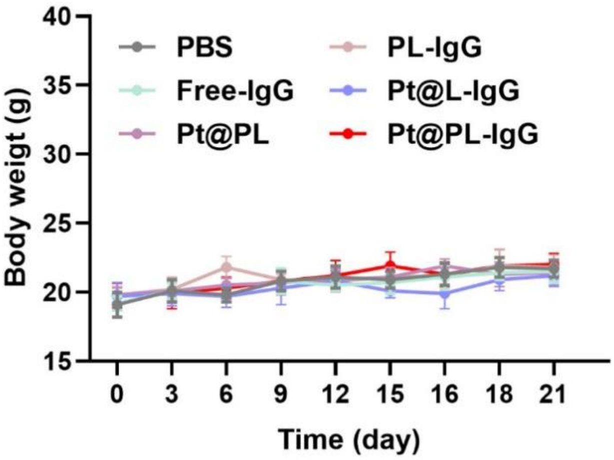

Figure S18. The body weight curves of disseminated lymphoma mice (n=5).

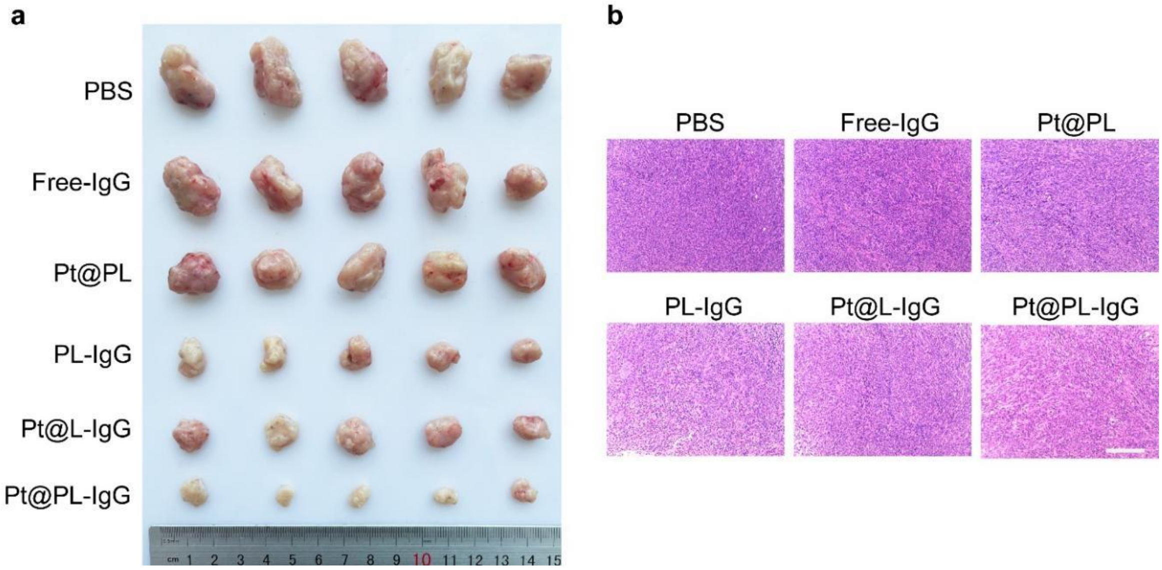

Figure S19. Anti-tumor efficacy of pancreatic cancer mouse models. **(a)** The photograph of *ex vivo* panc02 tumors in pancreatic cancer mice (n=5). **(b)** H&E staining of panc02 tumor tissues with different treatments. Scale bar, 200 μm.

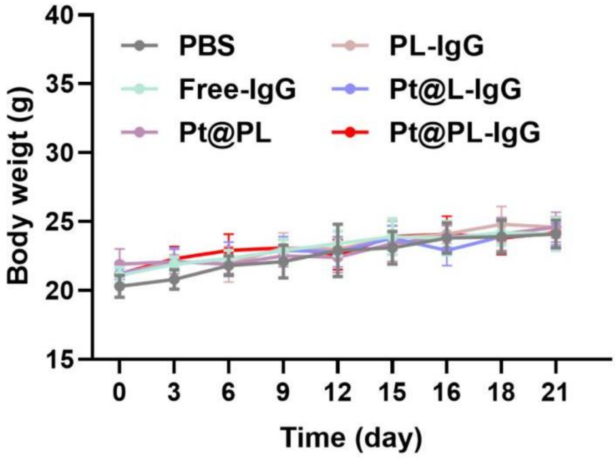

Figure S20. The body weight curves of Panc02 tumor-bearing mice (n=5).

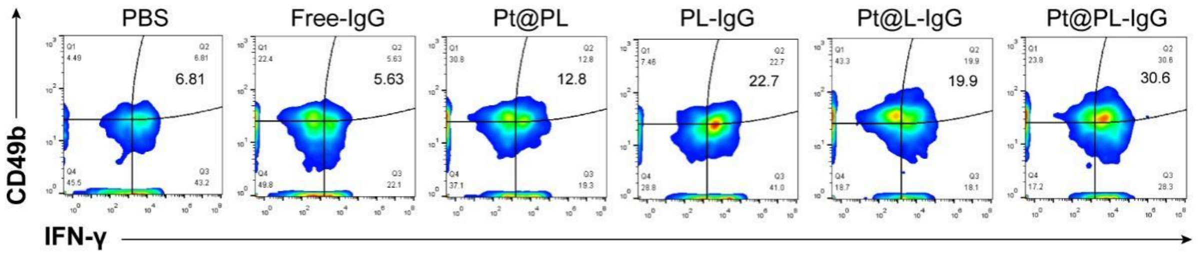

Figure S21. Representative flow cytometry plots of the population of IFN γ^+ NK cells in Panc02 tumors.

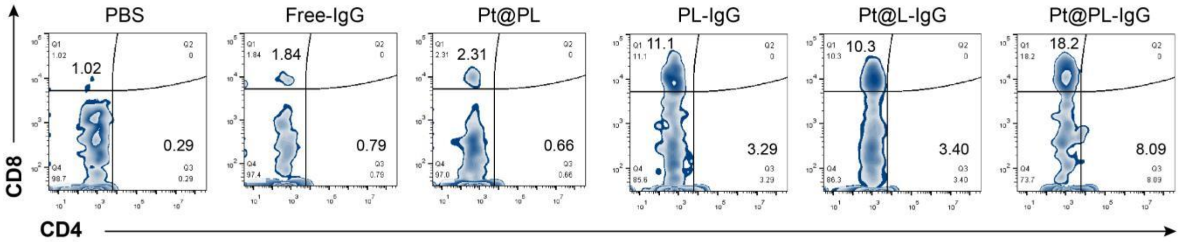

Figure S22. Representative flow cytometry plots of the effector T cells ($CD8^+$ T and $CD4^+$ T cells**)** in Panc02 tumors**.**

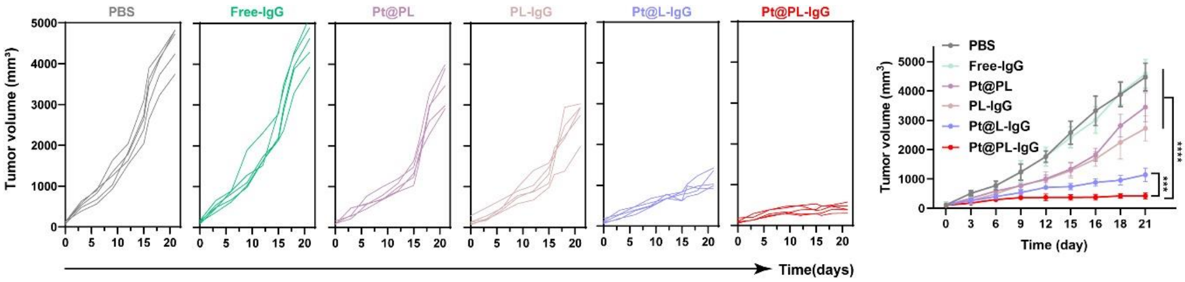

Figure S23. The B16-F10 tumors growth curves of mice with different treatments (n=5). Data are shown as mean ± SD; n represents the number of biologically independent samples. Student’s t-test. $^{*}P < 0.05$, $^{**}P < 0.01$ and $^{***}P < 0.001$ and $^{****}P < 0.0001$.

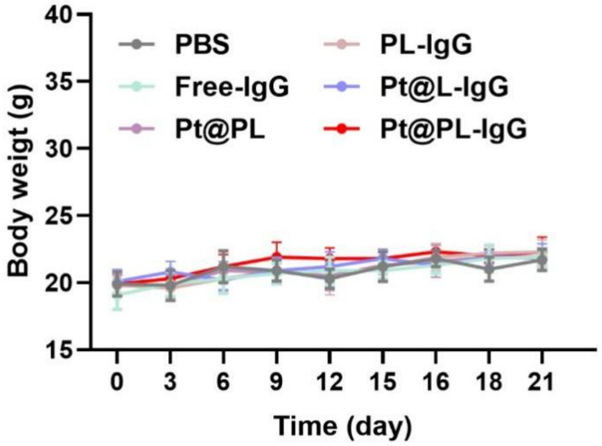

Figure S24. The body weight curves of metastatic melanoma mice (n=5).

Reference

[1] a) J. Yang, W. Liu, M. Sui, J. Tang, Y. Shen, *Biomaterials* **2011**, 32, 9136; b) C. Q. You, H. S. Wu, Z. G. Gao, K. Sun, F. H. Chen, W. A. Tao, B. W. Sun, *J. Mater. Chem. B.* **2018**, 6, 6752.
